# Rapid typing of infectious laryngotracheitis virus directly from tracheal tissues based on next-generation sequencing

**DOI:** 10.1007/s00705-022-05393-y

**Published:** 2022-03-04

**Authors:** Kinza Asif, Denise O’Rourke, Pollob Shil, Penelope A. Steer-Cope, Alistair R. Legione, Marc S. Marenda, Amir H. Noormohammadi

**Affiliations:** 1 Department of Veterinary Biosciences, Faculty of Veterinary and Agricultural Sciences, Asia-Pacific Centre for Animal Health, The University of Melbourne, Werribee, VIC 3030 Australia; 2 Research Services, Victoria University, Footscray, VIC 3011 Australia; 3 Department of Veterinary Biosciences, Faculty of Veterinary and Agricultural Sciences, Asia-Pacific Centre for Animal Health, The University of Melbourne, Parkville, 3010 Australia

## Abstract

Infectious laryngotracheitis virus (ILTV) is the causative agent of an economically important disease of chickens causing upper respiratory tract infection. Strains of ILTV are commonly identified by polymerase chain reaction restriction fragment length polymorphism (PCR-RFLP) and/or PCR high resolution melt (PCR-HRM) curve analysis targeting several genes. However, these techniques examine only a limited number of mutations present inside the target regions and may generate unreliable results when the sample contains more than one strain. Here, we attempted to sequence the whole genome of ILTV with known identity (class 9) directly from tracheal scrapings to circumvent *in vitro* culturing, which can potentially introduce variations into the genome. Despite the large number of quality reads, mapping was compromised by poor overlapping and gaps, and assembly of the complete genome sequence was not possible. In a map-to-reference alignment, the regions with low coverage were deleted, those with high coverage were concatenated and a genome sequence of 139,465 bp was obtained, which covered 91% of the ILTV genome. Sixteen single-nucleotide polymorphisms (SNPs) were found between the ILTV isolate examined and ILTV class 9 (JN804827). Despite only 91% genome coverage, using sequence analysis and comparison with previously sequenced ILTVs, we were able to classify the isolate as class 9. Therefore, this technique has the potential to replace the current PCR-HRM technique, as it provides detailed information about the ILTV isolates.

**Supplementary Information:**

The online version contains supplementary material available at 10.1007/s00705-022-05393-y.

Infectious laryngotracheitis (ILT) is an acute, highly contagious, and economically significant viral upper respiratory tract disease of chickens, occurring worldwide [7]. The causative agent is Gallid alphaherpesvirus 1*,* a member of the subfamily *Alphaherpesvirinae* and genus *Iltovirus* [7]. Genetic recombination between different strains is the driving force behind the evolution of herpesviruses [11, 21]. The rapid emergence of virulent recombinants highlights the need for reliable and timely strain identification for epidemiological investigations and timely control of the outbreak.

Commonly used ILTV typing techniques such as polymerase chain reaction restriction fragment length polymorphism (PCR-RFLP) [16] and PCR high resolution melt (PCR-HRM) curve analysis [6] rely on a limited number of genomic fragments that may not represent variations present outside the targeted regions [25]. Next-generation sequencing (NGS) can provide complete and accurate information about the identity of the ILTV strain involved. Many studies have determined partial or complete genome sequences of vaccine and field strains of ILTV from the USA [8, 27], China [17], and Australia [20, 22, 25] using isolation and propagation of the virus in chicken embryo kidney or fibroblast cells or in embryonated eggs, followed by purification of the virus using gradient centrifugation [2, 17, 20, 22, 26]. However, the possibility of genome modifications during the isolation and propagation process cannot be ruled out, and those occasionally may result in the failure of viral growth. Therefore, in this study, strain typing of ILTV was attempted directly from clinical tissues (tracheal scrapings) without *in vitro* or *in vivo* culturing to determine whether NGS can be used to accurately diagnose infection and identify the strain present.

All procedures involving animals were reviewed and approved by the University of Melbourne Animal Ethics Committee under approval number 1714358. Tracheal scrapings were collected from 5-week-old specific-pathogen-free chickens experimentally infected via eye-drop inoculation with the Australian ILTV class 9 isolate at a dose of 10^3.0^ PFU/bird. Tracheal scrapings were collected from two birds and pooled together in 3 mL of viral transport medium (VTM) containing medium 199 (Gibco) supplemented with 5% newborn calf serum (Invitrogen) and 1000 units of penicillin, 1000 µg of streptomycin, and 2.5 µg of amphotericin B per mL and stored at -80°C until further use. Viral DNA was extracted from tracheal scrapings using a QIAamp^®^ DNA Mini Kit (QIAGEN), following the manufacturer’s instructions. The quantity and quality of the DNA was determined using a Quantus Fluorometer (Promega) and microspectrophotometry (NanoDrop ND-1000, NanoDrop Technologies, Wilmington, DE), respectively. The quantity of DNA measured using the Quantus Fluorometer was 65 ng/µl. The presence of ILTV DNA in the tissue was confirmed using a PCR assay specific for the thymidine kinase gene (2240 bp amplicon size) [16].

DNA was sequenced using paired-end Illumina MiSeq 2 × 300 bp V3 chemistry at the Australian Genome Research Facility, Melbourne, Australia, using an Illumina Nextera Library Prep Kit. The overall quality of the reads was assessed using FastQC [1]. For pre-processing of Illumina reads, Fastp (Galaxy version 0.20.1) [3] was used for detection and removal of adapters and bases below a Phred quality score of 15. Initially, qScore 30 was used, which reduced the overall number of reads, which in turn reduced the number of reads mapped to the ILTV genome. Therefore, qScore 15 was used as a threshold. Sequencing reads were not trimmed for length. For taxonomic classification of the ILTV isolate, the nucleotide database was downloaded from NCBI in May 2020, dust masked for low-complexity regions, and converted into a Centrifuge (version 1.0.3) database, using the University of Melbourne High Performance Cluster, Spartan [18]. The quality of filtered reads was confirmed using FastQC [1]. Trimmed reads were used as input for *de novo* assembly using Unicycler (Galaxy version 0.4.8.0) [28]. Trimmed sequence reads were also mapped against the *Gallus gallus* reference genome sequence (GenBank no. NC_006088) using Bowtie2 mapper (version 2.3.0) [19] in Geneious Prime^®^ (version 2021.1.1) [15] to remove chicken-genome-associated reads from the data. Unmapped reads from this step were compared to the ILTV class 9 (JN804827) reference genome sequence using Bowtie2 in Geneious Prime^®^ with the parameters "end-to-end alignment" and "high sensitivity". Regardless of whether chicken DNA reads were removed or not, the number of reads mapped to the ILTV genome remained the same. Genome annotations were performed using “Transfer annotation” with a cost matrix of 93% similarity in Geneious Prime. A concatenated genome sequence was built from the regions where coverage was greater than a set threshold of “Highest Quality (60%)” in Geneious Prime. All sequence gaps were called as Ns and were deleted from the sequence. The Ns in the regions were removed, as the genome was being processed for phylogenetics and typing, and retaining the Ns would not have been informative in this context.

A multiple alignment of the concatenated sequence of the ILTV reisolate examined in this study with those of the other ILTV genomes (Table 1) was performed using MAFFT [14] with the FFT-NS-1 algorithm and a gap open penalty of 1.53 in Geneious Prime. Subsequent phylogenetic analysis was performed based on concatenated genomes using the neighbour-joining method with 1000 bootstrap replications in Geneious Prime. Illumina reads were mapped against the reference for screening of single-nucleotide polymorphisms (SNPs) with Find Variations/SNPs with FreeBayes (version 1.1.0-50-g61527c5) [9] in Geneious Prime using ILTV class 9 (JN804827) as a reference. Using a panel of six genomic regions (UL52, UL27, UL36, UL8, IR and US4) targeting SNPs as described previously [6], *in silico* analysis of the PCR-HRM products was performed. *In silico* PCR products were aligned to identify strain-specific SNPs to differentiate between the ILTV class 9 reisolate and other genotypes. DNA melt curves and denaturation profiles of the PCR products were analysed using uMelt (version 3.6.2 “Quartz”) [5] as described previously [6].

**Table 1:** Details of the complete genome sequences of ILTV isolates from the GenBank database used in this study

ILTV class	Strain name	GenBank accession no.	Genome size	Origin
Class 1	A20	JN596963	152978	Australia
Class 2	V1-99	JX646898	153630	Australia
Class 4	CSW-1	JX646899	151671	Australia
Class 7	Serva	HQ630064	152630	Australia
Class 7	7b	MN335811	152629	Australia
Class 8	ACC78	JN804826	152632	Australia
Class 9	CL9	JN804827	152635	Australia
Class 10		KR822401	152710	Australia

A total of 450,258 paired-end Illumina reads were obtained, with an average quality score of 37. After trimming, 444,694 paired-end reads were used. Taxonomic classification of Illumina reads using Centrifuge classified 66.2% (287,182) of the reads, with 98% of them (281,438) belonging to the *Gallus gallus* genome, 2% bacterial, and 0.5% viral. *De novo* assembly generated a total of 438 contigs ranging in size from 100 bp to 4728 bp that did not produce a complete genome sequence. Mapping of trimmed reads against the *Gallus gallus* genome (NC_006088) resulted in a total of 95,992 mapped reads and 348,702 unmapped Illumina short reads. Only 2746 out of 348,702 reads were mapped to the reference genome sequence of ILTV class 9, with a maximum depth of coverage of 18× and a mean depth of coverage of 4.3×. After removal of low-coverage/gap regions, the contig covered 91% of the ILTV genome. The estimated length of the concatenated sequence was 139,465 bp, and this sequence was compared with those of other ILTV genomes.

Sequence analysis of the ILTV reisolate from tracheal scrapings against Australian field and vaccine strains revealed a low degree of heterogeneity with all strains, with the highest nucleotide sequence identity to ILTV class 9 (Table 2). SNP analysis of the ILTV class 9 reisolate examined here referencing the published ILTV class 9 sequence (JN804827) revealed a total of 50 SNPs between the two, and 16 out of 50 SNPs had the same bases in all reads (Supplementary Table S1). Phylogenetic analysis showed that the ILTV reisolate clustered with the ILTV class 9 reference genome (Fig. 1). *In silico* analysis of the PCR-HRM products of six genomic regions (UL52, UL27, UL36, UL8, IR and US4) generated the same PCR-HRM pattern for the ILTV reisolate as class 9 (ABBBAA).

**Table 2:** Percent nucleotide sequence identity of the genome sequence of the ILTV isolate to previously published reference genome sequences

	ILTV isolate	Class 7b	Class 9	Class 8	Class 7 (Serva)	Class 10	Class 1 (A20)	Class 1 (SA2)
ILTV isolate		99.64	99.69	99.56	99.55	99.34	99.04	99.03
Class 7b	99.64		99.948	99.88	99.88	99.66	99.30	99.30
Class 9	99.69	99.94		99.87	99.86	99.64	99.35	99.34
Class 8	99.56	99.88	99.872		99.97	99.70	99.25	99.24
Class 7	99.55	99.88	99.861	99.97		99.69	99.23	99.22
Class 10	99.34	99.66	99.649	99.70	99.69		99.49	99.49
Class 1	99.04	99.30	99.354	99.25	99.23	99.49		99.97
Class 1	99.03	99.30	99.34	99.24	99.22	99.49	99.97

**Fig. 1:**
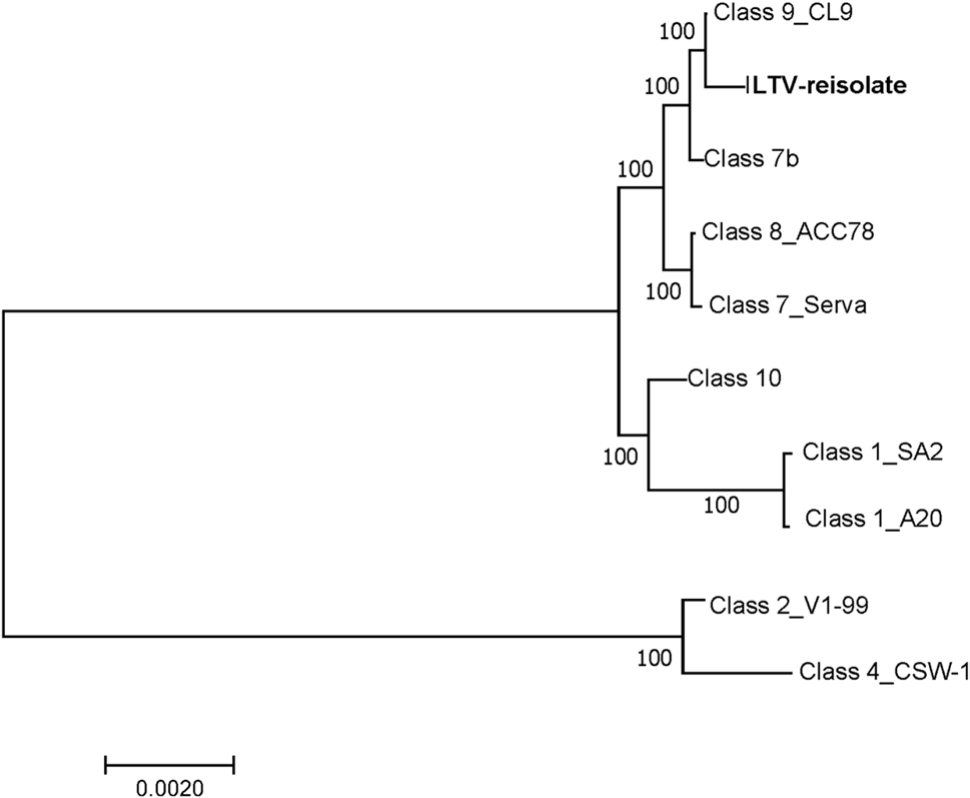
Phylogenetic tree based on concatenated genome sequences of ILTV reference isolates and the ILTV class 9 reisolate examined here, using the neighbour-joining method with 1000 bootstrap replicates. The length of the sequences used was 139,465 bp

Here, typing of an Australian ILTV class 9 reisolate was attempted directly from tracheal scrapings based on NGS without *in vitro* propagation. In theory, sequencing the genome of a pathogen directly from clinical samples reduces the risk of genome modifications that could occur after passage of the pathogen *in vitro* [4, 12, 13]. The sequence obtained directly from tracheal scrapings using Illumina sequencing generated reads of high quality; however, due to the small number of overlapping reads and the short length of the contigs, *de novo* assembly of the genome sequence was not possible. A possible explanation for the small number of reads might be a high host-to-pathogen genome copy ratio in the original sample, leading to fewer viral reads. It is noteworthy that the presence of mucus in the trachea may hinder the extraction of viral DNA, and the use of mucolytic agents such as N-acetyl-L-cysteine prior to extraction, as used for sputum samples [23] may help. Concatenation of the genome sequence after excluding low-coverage regions resulted in 91% genome coverage of the ILTV. Ideally, a complete genome sequence would have allowed full characterisation of the ILTV isolate in this study, but since the chief purpose of the study was to develop a strain typing technique that is more reliable than the currently available methods, directly from clinical specimens, that goal was still fulfilled. DNA capture and/or enrichment techniques such as the use of the SureSelect Target Enrichment System (Agilent Technologies) [10] were considered earlier in this project to increase the likelihood of obtaining a whole-genome sequence but were not pursued, as the cost appeared to be prohibitive for routine typing of ILTV from clinical specimens. Similarly, PCR amplification of the gaps followed by nucleotide sequencing could have been used to obtain a complete genome sequence of the ILTV isolate, but this was considered challenging, as there were several gaps throughout the genome.

The ILTV strain identification techniques currently in use, such as PCR-HRM [6] and PCR-RFLP [16], target multiple SNPs across different sites of the genome. The limitation of these techniques is that they do not detect SNPs outside the target regions. Therefore, direct sequencing from clinical samples would allow comprehensive analysis of the genome and accurate differentiation of ILTV strains [25]. Despite the coverage of the ILTV genome being only 91%, it was possible to classify it as class 9, and only 16 SNPs were detected in comparison to ILTV class 9 reference genome sequence. The fact that the reisolate used to experimentally infect the chickens from which the clinical samples were taken had the same genotype (JN804827) confirms the ability of this method to accurately genotype ILTV in clinical specimens. This also raises the possibility that genomic variations could have been introduced after isolation, subcloning, or *in vitro* propagation of ILTV isolates with published genome sequences.

Moreover, sequencing directly from clinical tissues circumvents the risk of producing chimeric genome sequences assembled from amplicons generated using multiple overlapping PCRs. Phylogenetic analysis based on the regions with sufficient coverage showed that the ILTV isolate clustered with class 9 isolates. Moreover, *in silico* analysis using six previously published gene sequences [6] gave essentially the same typing information and classified the ILTV reisolate as class 9 [6].

Although extensive research has been carried out on sequencing of herpesvirus genomes [24], there are no reports of attempts to sequence the ILTV genome directly from clinical samples. We propose that sequencing directly from clinical samples provides a promising means of typing ILTV and circumvents the need for *in vitro* culturing. Moreover, since only 0.6% of the Illumina reads (~450k) corresponded to ILTV, with a coverage of only 4.3× using the MiSeq platform, we propose that future studies should aim for ~4.5M reads in order to achieve a genome coverage of at least 30-fold. Further studies are needed to examine the efficiency of the technique using field samples, for example, where more than one ILTV strain may be present, or samples, where low levels of viral DNA are present due to the stage of infection or sample degradation.

## Supplementary Information

Below is the link to the electronic supplementary material.

Supplementary file1 (DOCX 17 kb)

Supplementary file2 (DOCX 21 kb)

## Data Availability

All data are supplied as a supplementary file.
