# Supplementary material for: Rapid typing of infectious laryngotracheitis virus directly from tracheal tissues based on next-generation sequencing
Source: Arch Virol. 2022 Mar 4;167(4):1151–5. doi: 10.1007/s00705-022-05393-y (PMC8964612; doi:10.1007/s00705-022-05393-y)
Supplement: Supplementary file 1 — Supplementary file1 (DOCX 17 kb) [file 705_2022_5393_MOESM1_ESM.docx]

| **Polymorphism Type** | **Nucleotide position** | **Change** | **Coverage depth** | **CDS** |
| --- | --- | --- | --- | --- |
| Substitution | 6011 | T -> C | 2 |  |
| Substitution | 7903 | A -> T | 4 | UL56 |
| Substitution | 10332 | T -> C | 2 | ORF-F |
| Substitution | 11286 | A -> T | 2 | UL54 |
| Deletion | 21230 | A -> - | 2 | UL48 |
| Substitution | 26111 | A -> G | 2 | ORF-A |
| Substitution | 30189 | A -> T | 2 | ORF-E |
| Substitution | 43998 | T -> A | 2 | UL29 |
| Substitution | 71721 | T -> A | 2 | UL39 |
| Substitution | 72485 | C -> T | 2 | UL41 |
| Substitution | 77580 | G -> A | 2 | UL44 |
| Substitution | 84085 | T -> A | 2 | UL19 |
| Substitution | 107433 | T -> A | 2 | UL3 |
| Substitution | 128865 | C -> T | 2 | US3 |
| Substitution | 140030 | A -> G | 2 | sORF4/3 |
| Substitution | 150346 | C -> T | 2 | ICP4 |
