# Supplementary material for: Rapid typing of infectious laryngotracheitis virus directly from tracheal tissues based on next-generation sequencing
Source: Arch Virol. 2022 Mar 4;167(4):1151–5. doi: 10.1007/s00705-022-05393-y (PMC8964612; doi:10.1007/s00705-022-05393-y)
Supplement: Supplementary file 2 — Supplementary file2 (DOCX 21 kb) [file 705_2022_5393_MOESM2_ESM.docx]

| **Polymorphism Type** | **Nucleotide position** | **Variant Nucleotide** | **Change** | **CDS** |
| --- | --- | --- | --- | --- |
| Substitution | 2661 | G | A -> G |  |
| Substitution | 3666 | G | C -> G |  |
| Substitution | 4568 | G | A -> G |  |
| Substitution | 6011 | C | T -> C |  |
| Substitution | 7903 | T | A -> T | UL56 |
| Substitution | 8232 | T | C -> T | ORF-F |
| Substitution | 8780 | A | T -> A | PRF-F |
| Substitution | 10083 | G | T -> G | ORF-F |
| Substitution | 10332 | C | T -> C | ORF-F |
| Substitution | 11286 | T | A -> T | UL54 |
| Substitution | 15301 | A | G -> A | UL52 |
| Substitution | 17174 | T | C -> T | UL51 |
| Deletion | 21230 | - | A -> - | UL48 |
| Substitution | 26111 | G | A -> G | ORF-A |
| Substitution | 30189 | T | A -> T | ORF-E |
| Substitution | 30510 | T | G -> T | ORF-E |
| Deletion | 33133 | - | G -> - | UL22 |
| Substitution | 39970 | T | C -> T | UL27 |
| Substitution | 40058 | G | A -> G | UL27 |
| Substitution | 42048 | G | A -> G | UL28 |
| Substitution | 43998 | A | T -> A | UL29 |
| Substitution | 51302 | T | A -> T | UL31 |
| Substitution | 51478 | G | A -> G | UL31 |
| Substitution | 61233 | C | T -> C | UL36 |
| Substitution | 71721 | A | T -> A | UL39 |
| Substitution | 72485 | T | C -> T | UL41 |
| Substitution | 77580 | A | G -> A | UL44 |
| Substitution | 84085 | A | T -> A | UL19 |
| Substitution | 87106 | A | G -> A | UL15 |
| Substitution | 87293 | T | C -> T | UL15 |
| Substitution | 87728 | A | G -> A | UL15 |
| Substitution | 87868 | G | A -> G | UL15 |
| Substitution | 89778 | G | T -> G | UL15 |
| Substitution | 89873 | T | G -> T | UL15 |
| Substitution | 95522 | T | C -> T | UL10 |
| Substitution | 96487 | C | T -> C | UL9 |
| Deletion | 97913 | - | A -> - |  |
| Deletion | 102990 | - | C -> - | UL6 |
| Substitution | 107433 | A | T -> A | UL3 |
| Substitution | 116317 | G | A -> G | ICP4 |
| Substitution | 123837 | T | A -> T | NCR |
| Substitution | 128678 | C | T -> C | US3 |
| Substitution | 128865 | T | C -> T | US3 |
| Substitution | 135900 | G | A -> G | US6 |
| Substitution | 136532 | A | T -> A | US7 |
| Substitution | 140030 | G | A -> G | sORF4/3 |
| Substitution | 145329 | T | G -> T | NCR |
| Substitution | 145338 | A | C -> A | NCR |
| Substitution | 149581 | A | G -> A | ICP4 |
| Substitution | 150346 | T | C -> T | ICP4 |
